# Highly Optimized Nitrogen-Doped MWCNTs through In-Depth Parametric Study Using Design of Experiments

**DOI:** 10.3390/nano9040643

**Published:** 2019-04-20

**Authors:** Alexander Plunkett, Katharina Kröning, Bodo Fiedler

**Affiliations:** 1 Institute of Advanced Ceramics, Hamburg University of Technology, 21073 Hamburg, Germany; 2 Institute of Polymer and Composites, Hamburg University of Technology, 21073 Hamburg, Germany; katharina.kroening@tuhh.de (K.K.); fiedler@tuhh.de (B.F.)

**Keywords:** N-CNT, pyridine, CVD, DoE, graphitization, Raman

## Abstract

The in-situ nitrogen doping of multiwalled carbon nanotubes via chemical vapor deposition is investigated employing design of experiments (DoE). The establishment of empirical DoE models allowed for the prediction of product features as a function of process conditions in order to systematically synthesize tailor-made nitrogen-doped carbon nanotubes. The high informative content of this approach revealed effects of individual parameters and their interaction with each other. Hence, new valuable insights into the effect of temperature, injection rate, and carrier gas flow on the doping level were obtained which give motivation to approach further theoretical studies on the doping mechanism. Ultimately, competitive nitrogen-doped carbon nanotube features were optimized and yielded promising combinations of achieved doping level, graphitization, and aspect ratios in comparison to present literature values.

## 1. Introduction

The outstanding mechanical, thermal, and electronic properties of carbon nanotubes (CNTs) have made them a subject of intensive research within the last decades [1,2]. Therefore, the quantity of published information regarding fabrication [3,4,5,6,7,8,9,10], characterization [11,12,13,14,15], functionalization [16,17,18,19], and possible applications [20,21,22,23,24,25] of single- and multiwalled carbon nanotubes (MWCNTs) is comprehensive. Among these topics, doping is a crucial strategy to modify the electronic behavior and surface reactivity of CNTs, expanding their application to the creation of flexible electronics [26], energy conversion/storage [22,27,28], devices, and catalysts [20,24,29]. The doping process involves the introduction of foreign atoms like nitrogen into the graphene lattice without decreasing any of the features that makes CNTs stand out. In this regard, and although many efforts have been made, optimal doping to allow for a sufficient length and structural integrity remains a challenge. Several studies on the issue [6,23,28,30,31] have shown an unfavorable, significant decrease of the graphitization through substitutional heteroatom doping. Since the graphitic π-bonding system provides electrical conductivity, a decrease of graphitization directly correlates with a diminishment of conductivity, therefore negatively impacting their use in conductive applications [29]. Furthermore, defects impair the growth of the CNTs, leading to a decreased total length and a reduced aspect ratio [6,28]. These features are important for specific applications as they promote percolation when used as fillers in composites [32,33] and increase the active surface area in catalysts [29]. It is therefore crucial to understand the influence of process parameters on the final properties in order to properly overcome these drawbacks.

A common approach to doping CNTs is the addition of a nitrogen-containing compound to the precursor mixture of the CNTs’ synthesis by chemical vapor deposition (CVD). Such methodology offers a flexible [4], low-cost [34], and scalable [3,34] solution, especially when compared to methods such as laser ablation [35], pyrolysis [36] or arc discharge [37]. The CVD process essentially consists of thermally decomposing a vaporized reaction feedstock to form elemental carbon which subsequently reorganizes in graphitic, tubular shape on a catalyst surface [34,38,39]. The CNT growth itself is initiated by elemental carbon saturating the catalyst until it segregates on the surface forming a graphene structure [38,40]. The catalyst usually consists of transition metal nanoparticles which are either on the target substrate or formed in situ during the reaction [41]. Brukh et al. [38] reported an insightful simulation of the decomposition process, where the decomposition proceeds via a radical chain reaction leading to elemental carbon as well as higher molecular compounds. The residence time in the reaction chamber mainly influenced the composition of the gas phase and thus the final product. This led to the conclusion that the process flow rates of the precursor determine the quality of the final CNTs and that, consequently, different carbon sources are expected to have different optimal flow rates.

Since the mechanism for obtaining pristine CNTs follows a cascade of complex reactions, the final formation of nitrogen-doped carbon nanotubes (N-CNTs) is still not completely understood. In order to gain insight into the mechanism, some parametric studies [30,31,42] investigated the influence of temperature and dopant concentration. Nevertheless, the study of the influence of other important factors (e.g., gas flow) has previously been limited to the synthesis of pristine CNTs. Furthermore, these factors are usually investigated by changing one factor at a time which limits the information to single parameter influences as well as the determination of optimal reaction conditions [43,44,45].

Herein, we present a comprehensive parametric study of the synthesis of N-CNTs to achieve targeted nitrogen contents with maximum graphitization and aspect ratios. Toluene and pyridine were used as precursors since our experience showed most promising results for compounds exhibiting aromaticity. Statistical design of experiments (DoE) was applied, a parametric approach to describing single experimental responses (in this case nitrogen content, graphitization, aspect ratio, and homogeneity) as polynomial functions of the investigated factors. The presented models give quantitative information about parameter influences and their interaction with each other. Furthermore, it leads to accurate predictions within the design space allowing for a systematical optimization of material properties with a minimum number of experiments [43,44,45,46].

## 2. Materials and Methods

### 2.1. Materials

Argon (Westfalen, Münster, Germany, 99.998%), ferrocene (Merck, Darmstadt, Germany, 98%), pyridine (Carl Roth, Karlsruhe, Germany ≥99%), toluene (Alfa Aesar, Karlsruhe, Germany, 99.5%), hydrogen (Westfalen, Münster, Germany, 99.999%) and quartz glass (Aachener Quarzglas-Technologie Heinrich, Aachen, Germany, 100 mm × 69 mm × 4 mm) were used as purchased without further treatment.

### 2.2. Synthesis of Nitrogen-Doped MWCNTs

**Figure 1:**
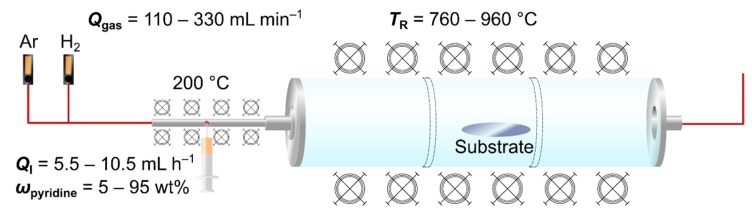
Schematic depiction of the used experimental setup and investigated process parameters.

Nitrogen-doped MWCNTs were synthesized by catalytic CVD using a modified procedure as reported previously [24]. A horizontal tube furnace HZS (Carbolite Gero, Neuhausen, Germany) with three heating zones and a quartz glass tube HSQ 300 (Aachener Quarzglas Technologie Heinrich, Aachen, Germany, Ø110 mm × 4 mm × 1100 mm) was used as a CVD reactor (Figure 1). A quartz glass was placed in the middle heating zone before heating the furnace to the desired reaction temperature *T*_R_ (760–960 °C). Ar and H_2_ gas in a ratio of 10:1 (*v/v*) were fed into a 200 °C heated stainless-steel evaporation unit (Ø = 6 mm) by a mass flow controller MV-302 and MV-392-H2 (Bronkhorst, Kamen, Germany) with a constant total gas flow (*Q*_gas_ = 110–330 mL min^−1^). The reaction feedstock consisted of 5 wt% ferrocene, and variable amounts of pyridine (*ω*_pyridine_ = 5–95 wt%) and toluene (i.e., no toluene for 95 wt% pyridine). The feedstock was injected using a syringe pump AL300 (World Precision Instruments, Friedberg, Germany) with a constant injection rate (*Q*_I_ = 5.5–10.5 mL h^−1^). After addition of 27.5 mL of the feedstock solution, the reaction was terminated by interrupting the precursor and hydrogen feed. The tube furnace was allowed to cool down under a constant argon gas flow of 400 mL min^−1^. The synthesized N-CNTs were removed from the substrate with a razor and investigated without further purification.

### 2.3. Sample Characterization

Carbon and nitrogen contents were determined by elemental analysis using a gas chromatograph coupled with a thermal conductivity detector CHNS macro analyzer vario MACRI cube (Elementar, Langenselbold, Germany). Iron content was determined by immersion of the samples in aqua regia and subsequent investigation using an inductively coupled plasma optical emission spectrometer. Thermogravimetric analysis (TGA) was carried out on a TGA Q500 (TA Instruments, New Castle, DE, USA) at 10 °C min^−1^ heating ramp under 60 mL min^−1^ gas flow of synthetic air (20% O_2_, 80% N_2_). Raman spectra were acquired using a Jobin Yvon HR800 (Horiba, Bensheim, Germany) quipped with a He-Ne laser (633 nm) and grid (600 mm^−1^) at 30 mW and 30 s integration time. Each sample was measured 5 times at different positions to provide average values. Electron microscopy images were acquired using either a Supra 55 VP FEG (Zeiss, Oberkochen, Germany) scanning electron microscope (SEM) at 5 kV or at a FEI Talos F200X (Thermo Fischer, Waltham, MA, USA) transmission electron microscope (TEM) at 200 kV acceleration voltage.

### 2.4. Design of Experiments and Model Calculation

**Figure 2:**
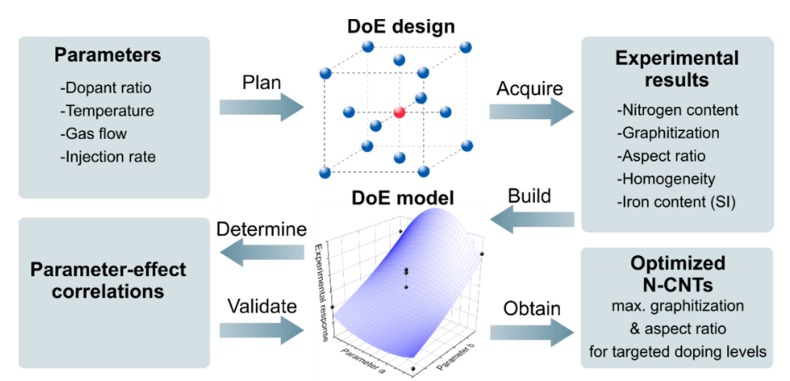
Schematic depiction of the applied optimization strategy leading to nitrogen-doped carbon nanotubes with different nitrogen contents and optimized graphitization and aspect ratios.

The general optimization strategy of this study is depicted in Figure 2. The experimental design, calculation of empirical models, and their evaluation were carried out using Design Expert 9 (Stat-Ease, Inc., Minneapolis, MN, USA). A face-centered central composite design covering 30 experiments including 6 replicates of the center point was applied to investigate the parameters listed in Table 1. Box-Cox power transformation [47] was employed to stabilize variance in case of heteroscedasticity and noted with the transformation factor *λ*. The calculated models were evaluated for statistical significance by analysis of variance (ANOVA) and validated by 10 additional confirmation tests.

## 3. Results and Discussion

**Figure 3:**
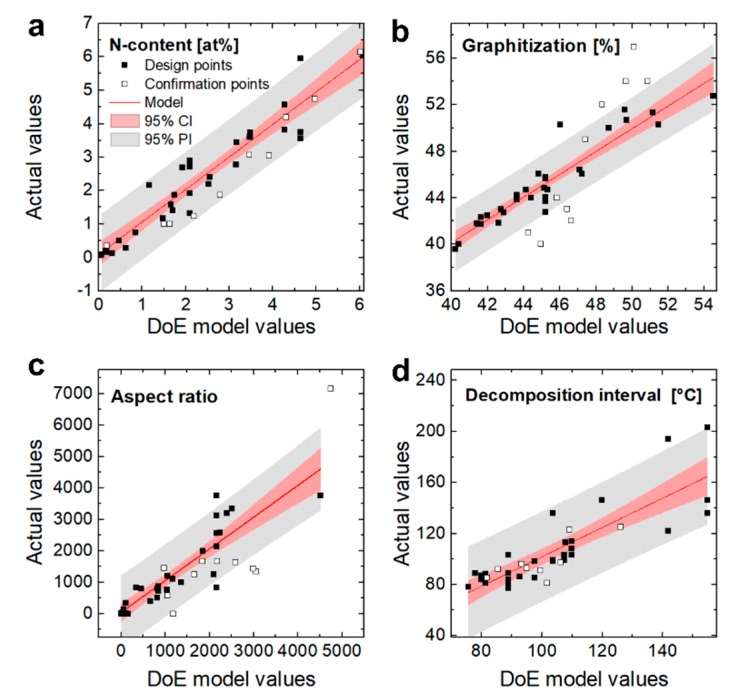
Comparisons between experimentally determined results and their theoretical values according to the DoE models (red line) for the nitrogen content (**a**), graphitization (**b**), aspect ratio (**c**), and the decomposition interval (**d**). Experiments used to establish the model are represented as black squares, while the ones used for model validation are indicated by white squares. Red and grey areas indicate the 95% confidence intervals (CIs) and prediction intervals (PIs), respectively.

As described in the experimental section, the N-CNTs were synthesized by adding pyridine as a nitrogen source to the toluene-based reaction feedstock. The amount of dopant source and the reaction temperatures are known to have a significant effect and were therefore chosen as factors. Additionally, the effects of both injection rate and carrier gas flow are known to have an effect on the residence time and the relative concentration of reactants [38,48]. Nevertheless, to the best of our knowledge, such parameters have not been taken into account in previous studies on CNT doping. Therefore, in this study, both parameters have been also considered. In order to provide a comprehensive overview of the N-CNT properties–parameter dependency, the relative nitrogen content, graphitization, aspect ratio, and the homogeneity (represented by the thermal decomposition interval [13]) of the N-CNTs were used as experimental responses for the applied DoE. This dependency was quantitatively described by empirical models which were fitted to the experimental results of a conducted preset of 25 different factor combinations (Table 1). The models were evaluated for statistical fit by ANOVA and confirmed by further confirmation experiments. The prediction accuracy of the DoE models is illustrated by comparing the DoE model values with the actual experimental data, which was used for the model establishment and its confirmation (Figure 3). Although the CVD process is known to be very sensitive to uncontrollable process conditions [49], the model predictions obtained are in good agreement with the experimental results. This is attributed to the statistical approach which allows an objective evaluation of effect to scatter relations [43,44,45]. For the sake of clearness, only discussion-relevant model plots are displayed graphically. Analogously, specific DoE model terms, their coefficient values, and ANOVA data can be found in the Supplementary Materials (SM) Section S3.

### 3.1. Nitrogen Content

**Figure 4:**
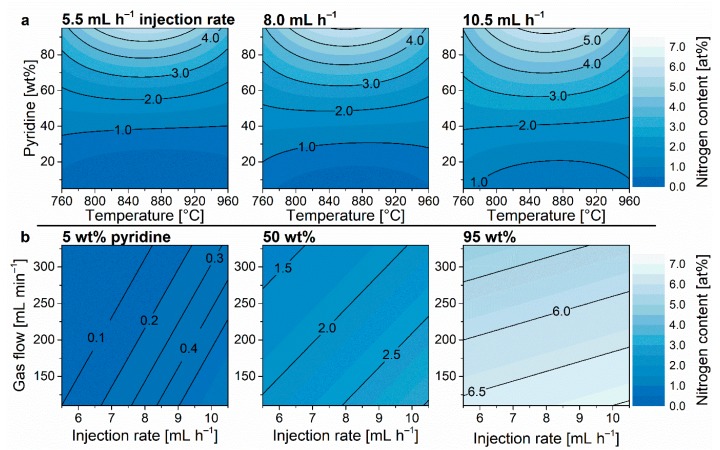
Model plots of nitrogen content in dependency of reaction temperature and pyridine ratio in the reaction feedstock for different injections rates (**a**) and in dependency of injection rate and carrier gas flow at various pyridine ratios (**b**).

**Figure 5:**
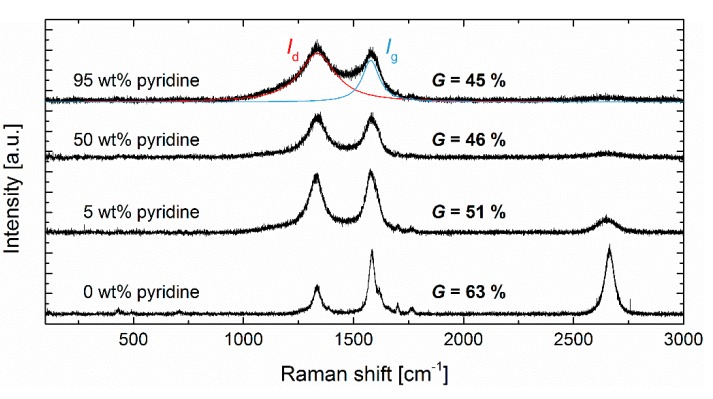
Transition of Raman spectra from neat to doped CNTs using different pyridine ratios. Signals of disordered and graphitic vibration modes are highlighted in red and blue, respectively.

**Figure 6:**
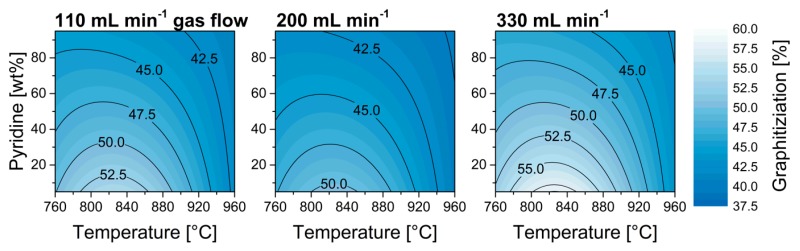
Model plots of graphitization in dependency of synthesis temperature and pyridine ratio in the reaction feedstock at various carrier gas flows.

**Figure 7:**
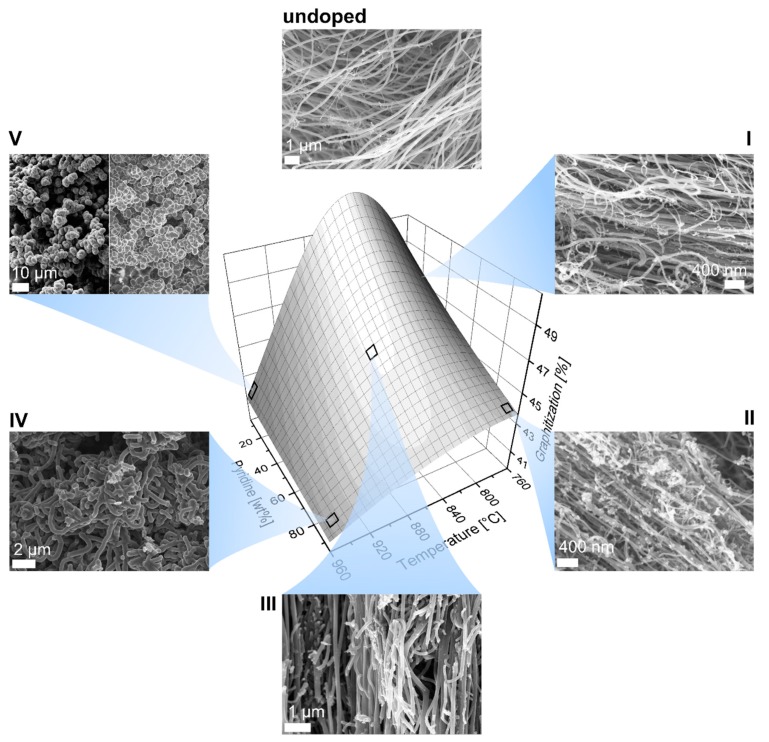
Surface response of graphitization versus temperature and pyridine content and SEM images of the N-CNTs obtained at different reaction conditions. The occurrence of branches and other optical defects acts in accordance with the established model of graphitization. TEM images can be found in Figure S3 (Supplementary Materials).

**Figure 8:**
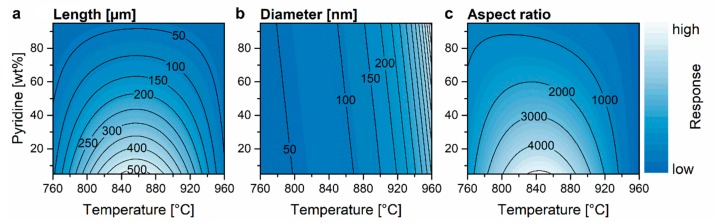
Model plots of N-CNT length (**a**), outer diameter (**b**) and aspect ratio (**c**) in dependency of synthesis temperature and pyridine ratio in the reaction feedstock.

**Figure 9:**
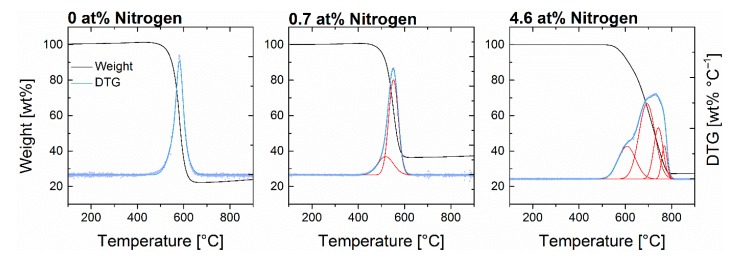
Thermogravimetric analysis of N-CNTs with various nitrogen content. Peak integration of the derivative thermogravimetric signals with Gauss functions reveals a signal broadening attributed to various decomposition processes.

**Figure 10:**
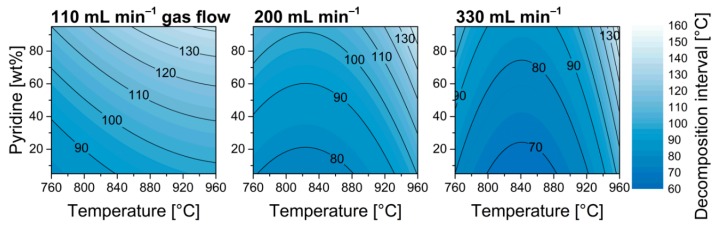
Model plots of the decomposition interval in dependency of synthesis temperature and pyridine ratio in the reaction feedstock for various carrier gas flows.

The nitrogen content of the synthesized N-CNTs was determined by elemental analysis of carbon (C) and nitrogen (N) and expressed as the atomic fraction x_N_ = n_N_/(n_N_ + n_C_). It was possible to variate the nitrogen content in the N-CNTs from 0.1 at% to 6.0 at% while featuring a good agreement between the model and the confirmation tests (Figure 3). For the discussion of single parameters’ effects (Figure 4, Figure 5, Figure 6, Figure 7, Figure 8, Figure 9 and Figure 10), the most relevant parameter interactions are visualized by contour plots of the DoE models. As expected, the pyridine–toluene ratio in the reaction feedstock had the most significant effect on the atomic composition of the N-CNTs. The visualization of the model in Figure 4a also confirms a significant temperature influence. Previous studies [6,30,42] generally assert that increasing temperatures diminish the nitrogen content which was attributed to C–C bonding being thermodynamically favored at higher temperatures. Surprisingly, the observations shown herein, however, show another trend. The results indicate that the influence of temperature on the nitrogen content is highly dependent on the observed area within the experimental space. This information was yielded since DoE provides a comprehensive determination of the cause–effect relationship, which is usually neglected in “one factor at a time” approaches [43,44,45]. As described above, the supply of the CNT building blocks carbon and nitrogen is the result of different reactions [38] which are affected by different parameters. Due to the different atomic compositions, pyridine and toluene undergo different decomposition reactions which occur at different temperatures [50,51]. We hence propose the assumption that the temperature influence on the atomic composition of N-CNTs is mainly due to the effect on the decomposition reactions of the precursors instead of the actual formation of the N-CNTs as assumed thus far. This assertion is also confirmed by the coded coefficient values in the ANOVA table (Table S1, SM) which allow for a direct, quantitative comparison of parameter effects [43,44,45]. The results show that the direct temperature terms have a very low significance (−0.03 and −0.09 for *T* and *T*^2^, respectively) compared to the effect of temperature–precursor interaction (−0.41 for the *T*^2^∙*ω*_pyridine_ term). Assuming the thermodynamic favorability of C–C bonding would be the main cause, one would rather expect a predominant direct influence of the temperature, i.e., *T* and *T*^2^ being significant which is not the case.

Furthermore, a completely new observation was provided by investigating the precursor injection rate and gas flow. To our knowledge, these factors have not been investigated for nitrogen doping of CNT thus far. Liu et al. [42] observed an influence of NH_3_ gas flow on the N-CNT doping level which is trivially reasoned by ammonia being the dopant source and thus a change of C and N ratio was obtained by changing this factor. In this study, however, the carbon and nitrogen sources were injected simultaneously as the reaction feedstock maintaining a constant ratio of the atoms in the gas phase as long as the feedstock composition was not changed. Nevertheless, a significant influence of injection rate and carrier gas flow on the final N-CNT composition can be seen in Figure 4b. Considering the contrary effect of each other, it can be assumed that the observed effect is caused by the change of absolute concentration of the N-CNT building blocks (i.e., an increase of injection rate and a decrease of gas flow both increase the concentration of particles in the gas phase). As described above, the CNT formation is initiated by carbon being saturated in the catalyst nanoparticles implying a limited carbon uptake rate. The amount of nitrogen could, however, still be increased at a higher nitrogen concentration in the gas phase. Another explanation can be given by considering kinetics. Herein, a higher rate constant and thus a higher concentration dependence would be the reason for the observed phenomenon. Nevertheless, the exact reason cannot be determined without further simulations. However, this observation could serve as valuable information for further studies on the understanding of the CNT doping mechanism.

### 3.2. Graphitization

Raman spectroscopy was applied to quantify the structural quality of the synthesized N-CNTs (Figure 5). The integrated intensity ratios *I*_d_/*I*_g_ of the disordered and graphitic carbon modes at 1330 cm^−1^ and 1580 cm^−1^ are in most cases used to represent the defect density [6,13,30,31,52]. For applying DoE, we defined the degree of graphitization according to *G* = *I*_g_/(*I*_d_ + *I*_g_) [53,54] since it led to statistically improved models. The used quadratic polynomial terms sufficiently agree with the design points while the confirmation tests are located significantly beyond the 95% prediction interval (Figure 3). The quite pronounced lack of fit (Table S2, SM) indicates that the resolution of the design is not high enough to precisely represent the effects in the whole design space. Therefore, the model established is not able to predict exact values but still provides considerable information about parameter effects and serves as a guide for optimization.

In previous studies, a relation between nitrogen doping and increasing loss of structural integrity was observed [6,23,28,30,31]. Nevertheless, distinctive parameter dependencies allow for a separate adjustment of these properties in some parts of the experimental space. The model features a strong quadratic temperature effect leading to optimum reaction temperatures with regards to graphitization at 800–820 °C (Figure 6). Furthermore, a strong quadratic effect was also observed for the carrier gas flow leading to a minimum of graphitization at 200 mL min^−1^, despite the nitrogen inclusion showed a linear dependency for this parameter, i.e., one would expect the lowest graphitization at the lowest gas flow based on the results shown in Figure 4. This phenomenon can be attributed to optimal reaction conditions for decomposing the carbon source or catalyst precursor [38].

The surface response of graphitization versus temperature and pyridine content in Figure 7 can be used as a qualitative comparison of the obtained morphologies and specific graphitization. Undoped MWCNTs with 63% graphitization featured predominantly straight, parallelly growing arrays with little occurrence of defects in terms of kinks. The addition of 5 wt% pyridine into the reaction feedstock (Figure 7I) already caused strong curvatures of the CNTs and the appearance of nontubular structures which further increased at higher pyridine ratios up to 95 wt% (Figure 7II). At higher temperatures (Figure 7III), mostly straightly growing structures were observed which however exhibited branching and secondary growing. The branching increased with further rise in temperature and pyridine ratio (Figure 7IV) until no straight-lined CNTs could be detected. At high temperatures and low pyridine ratios (Figure 7V), no tubular structures were observed. Raman spectroscopy confirmed typical graphene modes that can be associated to agglomerated carbon black.

### 3.3. Aspect Ratio

The outer diameter of the synthesized doped MWCNTs was determined by image analysis of the SEM images measuring >100 single CNTs. The results were divided in classes of 5 nm size and investigated regarding to their relative frequency. It turned out that more than half of the samples exhibited bimodal size distributions of which only one mode was predominant. The class with the highest relative frequency was thus used for the model to accomplish a representative average. The length was determined based on the forest length of fully imaged, continuous CNT bundles. Strongly branched CNTs often did not yield any complete bundles and were therefore tagged with a standard value of 5 µm. This value gives an underestimation of the actual average length; nevertheless, a representative value was essential for the statistical analysis and its accuracy was not significantly affecting the resulting model due to its low magnitude relative to other samples. Soot-like particles were treated the same way.

Model plots for the N-CNT dimensions are shown in Figure 8. A clear correlation between the length and the graphitization appears (compare Figure 6). Such a correlation can be related to the fact that defects impair the growth of CNTs. This compliance benefits the simultaneous optimization of the properties. Hence, the model would allow achieving N-CNTs with aspect ratios higher than 10^3^ which is in the same order of magnitude as undoped CNTs optimized in the herein used system [23,33,55]. The significant effect of growth temperature on the CNT diameter was also observed in previous studies [6,30,31] and can be explained by an increased amorphous carbon shell around the catalyst particles due to faster segregation leading to higher amounts of CNT walls [40].

### 3.4. Homogeneity

It is well known that the quality of single CNTs within a batch can highly scatter and nontubular structures are often observed [13,56] (Figure S5, SM); however, the homogeneity of an obtained CNT sample is generally not discussed in publications. It can be expected that homogeneity has a significant impact when it comes to application of CNTs since cleaning of the crude product is limited and leads to additional costs. In this study, TGA was used to evaluate the thermal stability of a sample and to obtain information about impurities [13]. While undoped CNTs featured a sharp and symmetric decomposition curve, doping of the CNTs led to broader peaks of the derivative thermogravimetric (DTG) signals (Figure 9) that can be associated to increased occurrence of different decomposition processes as a consequence of inhomogeneity. The degree of homogeneity was quantified by the decomposition interval Δ*T*_d_ which is the temperature range in which 5–95 wt% of the sample decomposes. The reproducibility of the chosen value was limited due to high scatter and does not allow for predictions. Nevertheless, the statistical analysis provides a quite significant model and hence sufficiently represents the parameter effects.

The highest homogeneities were achieved in a temperature range of 800–850 °C (Figure 10). Temperatures above these values led to a strong drop in homogeneity which is attributed to the occurrence of morphologically altered structures as shown before (Figure 7). The negative influence of the pyridine ratio could indicate an inhomogeneous distribution of nitrogen inclusion into the CNTs which reflects a general issue in doping of nanomaterials [57]. The positive effect of carrier gas flow can be explained by a shorter residence time of the precursor in the reaction space leading to fewer high-molecular compounds [38].

### 3.5. Optimized N-CNTs

The results confirmed the general conflict between nitrogen doping and morphological properties of CNTs. The herein established empirical models can be used in order to analytically determine compromise solutions for N-CNT properties. This was demonstrated by calculating parameter combinations leading to N-CNTs with optimized graphitization and aspect ratios at different nitrogen doping levels. Hence, nitrogen levels of 0.3–6.1 at% were synthesized (Table 2). A comparison of the parameter combination for 4.7 at% and 6.1 at% samples once again highlights the significant effect of carrier gas flow reduction on the nitrogen content since other parameters vary only slightly. Furthermore, the iron content *ω*_Fe_ originating from the remaining catalyst was determined (The respective DoE model is discussed in the SM Section S1).

Most of the optimized N-CNTs were obtained in high yields of >1 g (corresponding to >7 mg cm^–2^) and high aspect ratios of >1000. Special emphasis should be placed on the sample containing 0.3 at% nitrogen which achieved an aspect ratio of ~7000. This value reaches the same order of magnitude as undoped CNTs being previously optimized in the used system by Mecklenburg et al. [55].

**Figure 11:**
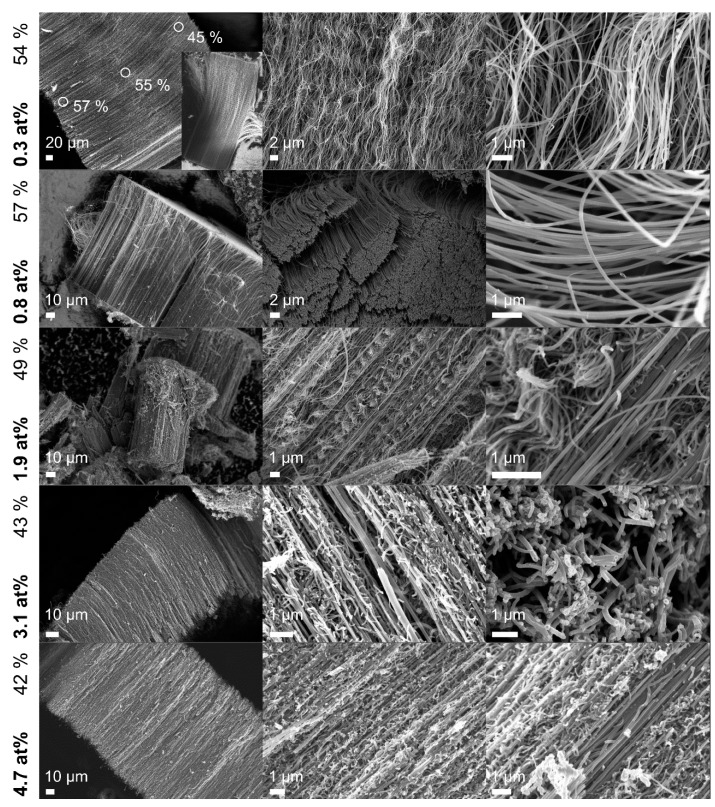
SEM images of optimized N-CNTs with various nitrogen contents and graphitization. N-CNTs containing 0.8 at% nitrogen feature an exceptionally smooth morphology. For very long N-CNTs, i.e., containing 0.3 at% nitrogen, a gradual increase of waviness with increasing CNT length was observed.

The N-CNTs were predominantly obtained as straight-growing arrays, as shown in Figure 11 (TEM images are shown in Figure S4 in the SM). The increasing nitrogen content then again led to a rise in waviness and branching. Compared to the previous samples used for the DoE shown in Figure 7, the optimized N-CNTs highlight a remarkable optical improvement of the morphology. The optimization was particularly pronounced for doping levels up to 2 at%. The 0.8 at% sample yielded the highest graphitization of 57% featuring an exceptionally smooth surface.

A noticeable increase of waviness within an N-CNT array was observed for the sample exceeding a total length of ~140 µm (cf. 0.3 at% nitrogen). The size of the CNTs allowed for a spatially resolved Raman analysis at different array heights and revealed a decrease of graphitization from 57% to 45% with the increasing length of the N-CNTs. This phenomenon could indicate an increasing probability of defects being introduced with regards to the total length or doping level of the N-CNTs.

**Figure 12:**
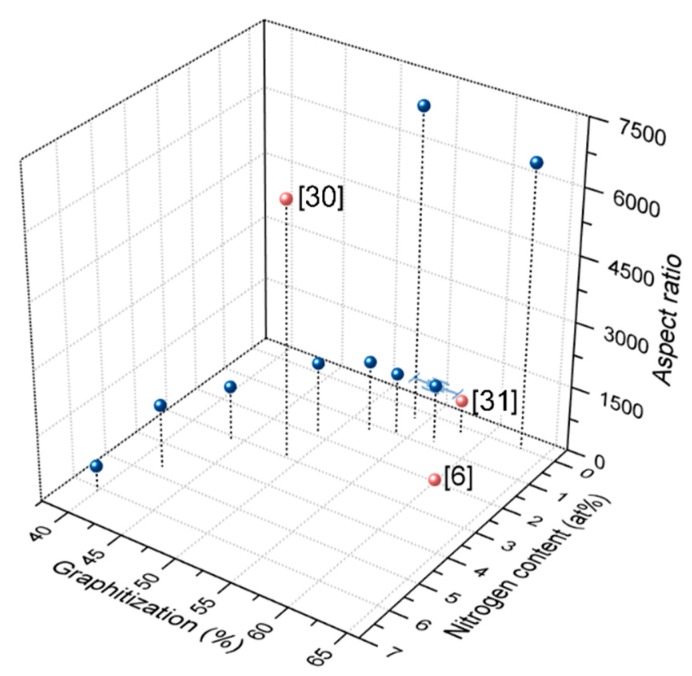
Graphitization and aspect ratios of optimized N-CNTs compared with those in previous reports [6,30,31].

A comparison with previous studies on N-CNTs [6,23,24,30,31,43,52,56,58,59,60,61,62,63] revealed that significantly higher values for the combination of graphitization and aspect ratio have been achieved in this study. Values in the same regime of graphitization were reported in two other publications [6,31], however, showing lower aspect ratios in the respective doping level (Figure 12). Yadav et al. [30] reported a high aspect ratio of ~6000 and a graphitization of 49% (derived from *I*_d_/*I*_g_ = 1.03) at a doping level of 3 at%. Nevertheless, the nitrogen content was determined by X-ray photoelectron spectroscopy and is hence not comparable to values of the herein used quantification technique.

## 4. Conclusions

The competition between doping level, graphitization, and resulting aspect ratio was figured out to optimized process conditions and can be used as a toolbox to obtain excellent N-CNT characteristics for targeted doping levels which outstand most reported features thus far. Due to applying DoE, the established empirical models predict the nitrogen content of the N-CNTs in a wide range with a relative high precision. Simultaneously, the graphitization, aspect ratio, and homogeneity was tuneable with satisfying confidence intervals despite the challenge of CVD process control [49]. This allowed us to achieve a high yield of optimized N-CNTs enabling a wide field of further investigations (e.g., application in electronics, energy conversion/storage, and catalysts). The profound informative output of the DoE models extended the observations of former studies [6,30,42] regarding the temperature influence on the nitrogen inclusion and assisted in the establishment of new assumptions. Furthermore, the effects of carrier gas flow and injection rate on the doping level were presented and can be useful in further theoretical studies to clarify the doping mechanism of CNTs.

## Figures and Tables

**Table 1:** Investigated reaction parameters and used values for each respective coded step. The inset shows the applied face-centered central composite design highlighting the used parameter combinations (blue/red spheres) in the present parameter space leading to overall 30 experiments.

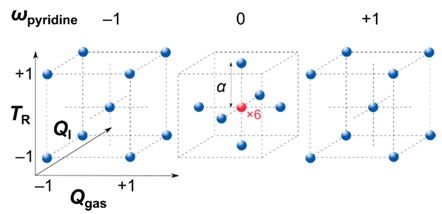				
Coded Value	*T*_R_	*ω*_pyridine_	*Q*_gas_	*Q*_I_
	[°C]	[wt%]	[mL min^−1^]	[mL h^−1^]
−1	760	5	110	5.5
+1	960	95	330	10.5
−*α*	760	5	110	5.5
+*α*	960	95	330	10.5

**Table 2:** Process parameters for optimized N-CNTs featuring various nitrogen contents and optimized graphitization and aspect ratios. The standard deviation shown for the sample containing 0.8 at% nitrogen is based on four replicates in order to represent the replicability of optimized values.

Measured Properties					Input Values			
*x*_N_	*G*	*I*_d_/*I*_g_	*η*	*ω*_Fe_	*ω*_pyridine_	*Q*_gas_	*Q*_I_	*T*_R_
[at%]	[%]			[wt%]	[wt%]	[mL min^−1^]	[mL h^−1^]	[°C]
undoped	63	0.60	6500	4.9	0.0	220	5.5	760
0.3	54	0.86	7143	2.3	5.0	175	7.4	843
0.8 ± 0.2	57 ± 2	0.77 ± 0.05	1333 ± 63	6.3 ± 0.5	22.0	110	10.5	820
1.0	54	0.87	1429	5.2	25.6	110	10.5	826
1.2	52	0.95	1625	5.0	36.3	110	10.5	834
1.9	49	1.04	1667	5.0	46.1	110	10.5	824
3.1	44	1.06	1250	5.6	62.1	110	10.5	831
4.7	42	1.40	593	3.5	95.0	330	5.5	828
6.1	40	1.48	888	3.7	93.0	110	5.5	823

## Supplementary Materials

The following are available online at https://www.mdpi.com/2079-4991/9/4/643/s1, Figure S1. Comparison between experimentally determined results and their theoretical values according to the DoE model, Figure S2. Model plots of iron content in dependency of reaction temperature and pyridine ratio in the reaction feedstock for different injection rates, Figure S3. TEM images of undoped and N-CNTs used for the morphology comparison in Figure 7, Figure S4. TEM images of optimized N-doped MWCNTs at different nitrogen contents, Figure S5. SEM images illustrating the inhomogeneity within two N-CNT samples with different decomposition intervals, Δ*T*_d_, Table S1. ANOVA data of the design concerning the N-CNTs’ nitrogen content, Table S2. ANOVA data of the design concerning the N-CNTs’ graphitization, Table S3. ANOVA data of the design concerning the N-CNTs’ aspect ratio, Table S4. ANOVA data of the design concerning the N-CNTs’ decomposition interval, i.e., homogeneity, Table S5. ANOVA data of the design concerning the N-CNTs’ Fe content.
