# Supplementary material for: Highly Optimized Nitrogen-Doped MWCNTs through In-Depth Parametric Study Using Design of Experiments
Source: Nanomaterials (Basel). 2019 Apr 20;9(4):643. doi: 10.3390/nano9040643 (PMC6523270; doi:10.3390/nano9040643)
Supplement: Supplementary file 1 [file nanomaterials-09-00643-s001.pdf]

nanomaterials

Supplementary Materials (SM)

Highly Optimized Nitrogen-Doped MWCNTs through In-Depth Parametric Study Using Design of Experiments

Alexander Plunkett [1,*], Katharina Kröning [2] and Bodo Fiedler [2]

1 Institute of Advanced Ceramics, Hamburg University of Technology, 21073 Hamburg, Germany
2 Institute of Polymer and Composites, Hamburg University of Technology, 21073 Hamburg, Germany; katharina.kroening@tuhh.de (K.K.); fiedler@tuhh.de (B.F.)
* Correspondence: alexander.plunkett@tuhh.de

Further sample characteristics and ANOVA data for evaluated designs.

S1. N-CNT purity

The purity of the N-CNTs was determined by measuring the iron content via elemental analysis. The obtained values ranged from 0.3 wt% to 13.4 wt% and can be represented by a very significant model as shown in **Figure S1**.

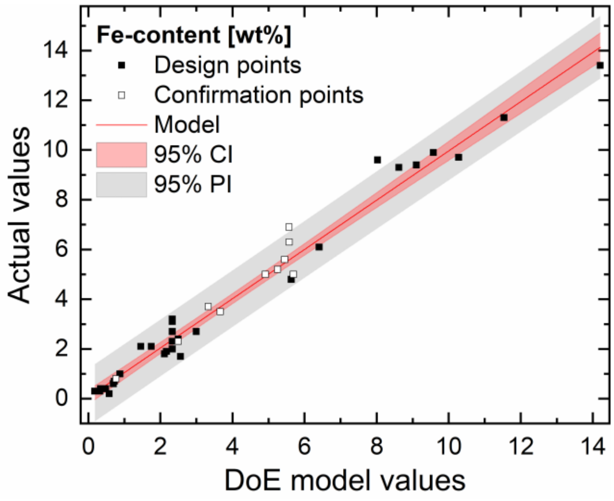

Figure S1. Comparison between experimentally determined results and their theoretical value according to the DoE model (red line). Experiments used to establish the model are represented as black squares, while the ones used for model validation as white squares. Red and grey areas indicate the 95 % confidence (CI) and prediction intervals (PI), respectively.

The reaction temperature was found to have the highest effect on the remaining N-CNTs (**Figure S2**). This can be attributed to the significant increase of N-CNT diameter (cf. **Figure 8**), i.e. increased number of N-CNT walls per catalyst particle. The coded coefficient values in **Table S5** indicate that iron content is promoted with decreasing carrier gas flow and increasing injection rate, likely due to the increase of catalyst precursor concentration in the gas phase.

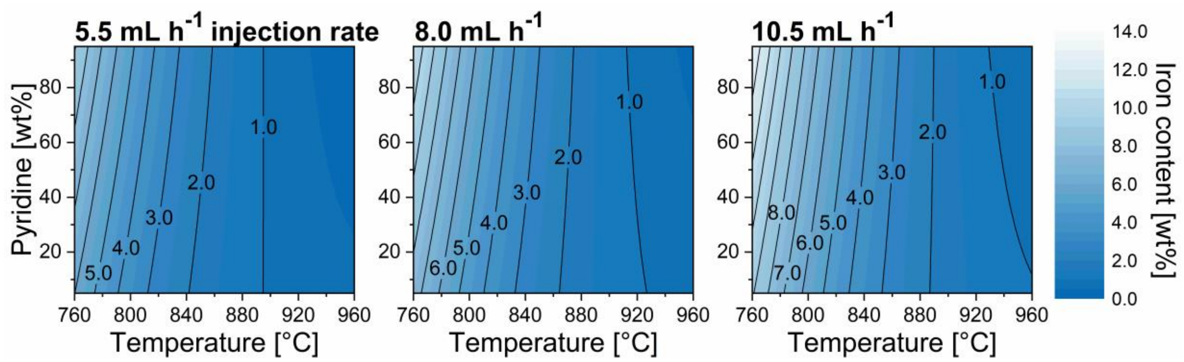

Figure S2. Model plot of iron content in dependency of reaction temperature and pyridine ratio in the reaction feedstock for different injections rates.

S2. Transmission electron microscopy images

In order to provide a further insight to the nanostructure of the exemplary samples used for the discussion of the morphology changes in **Figure 7**, TEM images are shown in **Figure S3**.

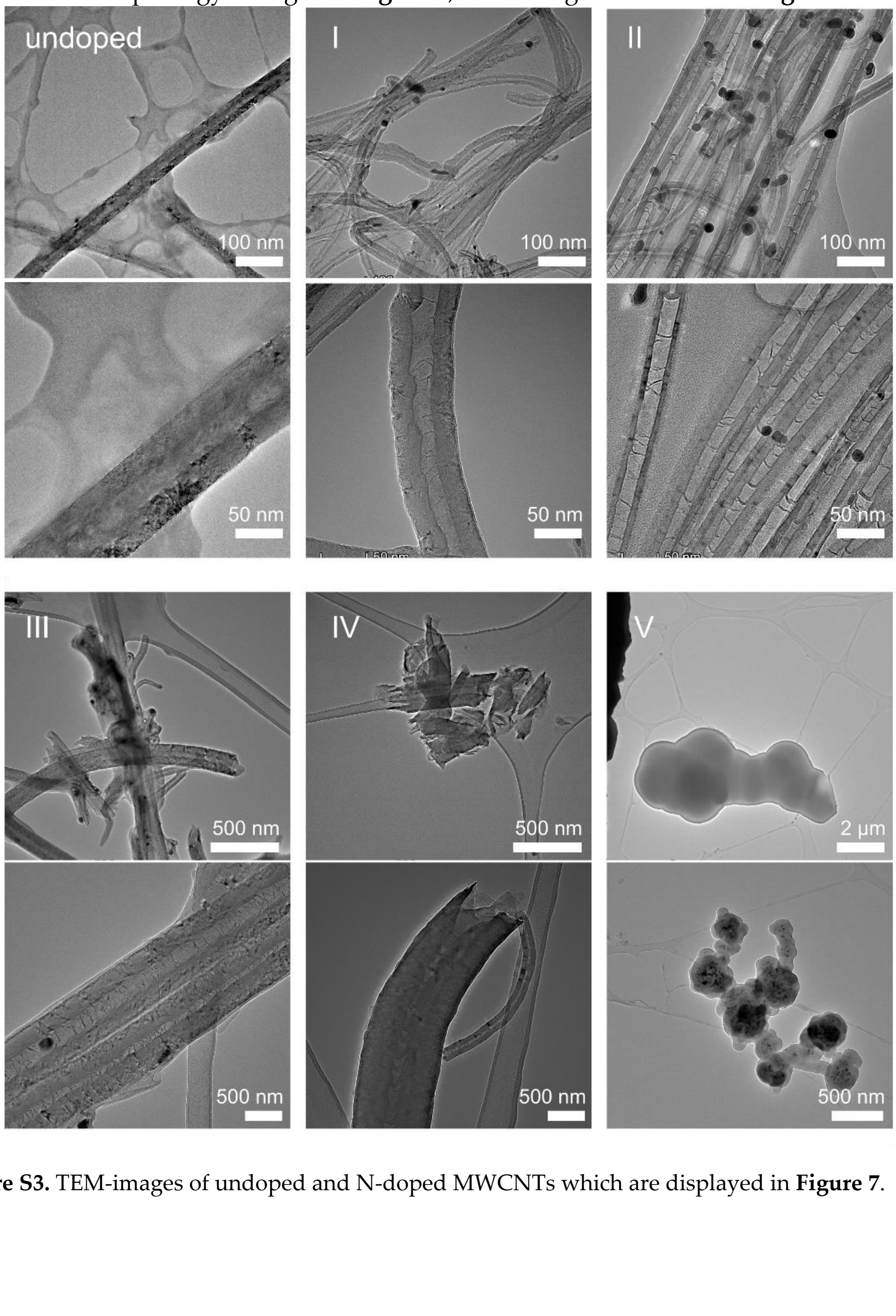

Figure S3. TEM-images of undoped and N-doped MWCNTs which are displayed in **Figure 7**.

The optimized N-CNTs featured typical morphological alteration of the wall structure: Transmission electron microscopy images show predominantly mesh-like structure at ~1 at% nitrogen (**Figure S4**). Higher doping levels led to a rise of bamboo structures whose segment distances decreased with increasing nitrogen content. All optimized N-CNTs possessed 20–130 walls with an interwall distance of 3.5 Å. The CNT tips were mostly present in a close condition.

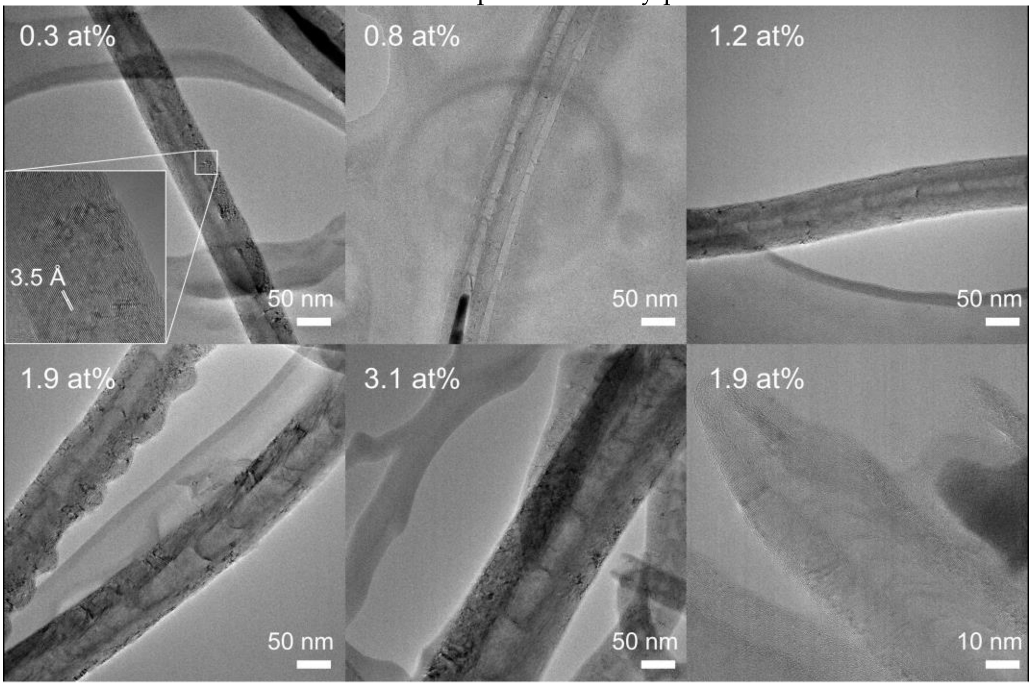

Figure S4. TEM-images of optimized N-doped MWCNTs at different nitrogen contents. A typical mesh-like and bamboo structure was observed for all samples.

S3. ANOVA Tables

Table S1. ANOVA data of the design concerning the N-CNTs′ nitrogen content. For each polynomial term the *F* and *p* value is shown as well as the coded [−1/+1] and actual values of the respective coefficients. The used transformation for the fit is given by *λ*.

Term	*F* value	*p* value	coded	actual
$\lambda = 0.5$				
Model	31.55	<0.0001	1.44	27.15
T_R	0.56	0.4632	−0.03	−0.06
Q_I	11.62	0.0025	0.15	0.11
Q_{gas}	10.32	0.0040	−0.14	1.31×10^{-3}
$\omega_{pyridine}$	52.90	<0.0001	1.02	−0.65
$T_R\ \omega_{pyridine}$	0.52	0.4797	0.03	1.57×10^{-3}
$Q_I\ \omega_{pyridine}$	5.35	0.0305	−0.11	-9.77×10^{-4}
T_R^2	1.49	0.2358	−0.09	3.63×10^{-5}
$T_R^2\ \omega_{pyridine}$	7.58	0.0116	−0.41	-9.08×10^{-7}
LOF	0.55	0.8392		

Table S2. ANOVA data of the design concerning the N-CNTs' graphitization. For each polynomial term the *F* and *p* value is shown as well as the coded [−1/+1] and actual values of the respective coefficients. The used transformation for the fit is given by λ.

Term	*F* value	*p* value	coded	actual
$\lambda = -3$				
Model	24.51	<0.0001	1.08×10^{-5}	2.84×10^{-4}
T_R	107.08	<0.0001	2.14×10^{-6}	-6.90×10^{-7}
Q_I	8.05	0.0096	5.87×10^{-7}	2.35×10^{-7}
Q_{gas}	10.03	0.0045	-6.55×10^{-7}	6.68×10^{-8}
$\omega_{pyridine}$	17.27	0.0004	2.69×10^{-6}	-2.43×10^{-6}
$T_R\ \omega_{pyridine}$	7.22	0.0135	-5.86×10^{-7}	5.92×10^{-9}
T_R^2	23.80	0.0001	2.41×10^{-6}	4.17×10^{-10}
Q_{gas}^2	16.37	0.0005	-2.00×10^{-6}	-1.65×10^{-10}
$T_R^2\ \omega_{pyridine}$	5.36	0.0304	-1.58×10^{-6}	-3.52×10^{-12}
LOF	1.26	0.4102		

Table S3. ANOVA data of the design concerning the N-CNTs' aspect ratio. For each polynomial term the *F* and *p* value is shown as well as the coded [−1/+1] and actual values of the respective coefficients. The used transformation for the fit is given by λ.

Term	*F* value	*p* value	coded	actual
$\lambda = 0.5$				
Model	8.33	0.0001	46.60	-3.66×10^{3}
T_R	32.23	<0.0001	−12.21	8.58
Q_I	2.61	0.1249	−3.47	25.87
Q_{gas}	0.11	0.7478	−0.70	1.34
$\omega_{pyridine}$	9.55	0.0066	−20.62	32.33
$T_R\ Q_I$	0.43	0.5222	1.48	-3.21×10^{-2}
$T_R\ Q_{gas}$	0.64	0.4361	−1.81	-1.55×10^{-3}
$T_R\ \omega_{pyridine}$	2.48	0.1340	3.58	-7.70×10^{-2}
$Q_I\ Q_{gas}$	0.00	0.9705	0.09	−0.15
$Q_I\ \omega_{pyridine}$	0.06	0.8146	0.54	4.81×10^{-3}
$Q_{gas}\ \omega_{pyridine}$	0.52	0.4792	−1.64	-3.32×10^{-4}
T_R^2	55.24	<0.0001	−26.51	-4.91×10^{-3}
$T_R\ Q_I\ Q_{gas}$	4.37	0.0520	4.75	1.73×10^{-4}
$T_R^2\ \omega_{pyridine}$	8.33	0.0103	20.34	4.52×10^{-5}
LOF	0.6	0.7804		

Table S4. ANOVA data of the design concerning the N-CNTs' decomposition interval, i.e. homogeneity. For each polynomial term the *F* and *p* value is shown as well as the coded [−1/+1] and actual values of the respective coefficients. The used transformation for the fit is given by λ.

Term	*F* value	*p* value	coded	actual
$\lambda = 0.5$				
Model	12.52	<0.0001	1.13×10^{-3}	0.20
T_R	19.30	0.0002	-1.22×10^{-3}	-4.35×10^{-4}
Q_{gas}	5.13	0.0328	1.99×10^{-3}	-1.36×10^{-3}
$\omega_{pyridine}$	32.64	<0.0001	-1.60×10^{-3}	-3.55×10^{-5}
$T_R\ Q_{gas}$	0.46	0.5050	-2.00×10^{-3}	3.23×10^{-6}
T_R^2	12.90	0.0015	-1.68×10^{-3}	2.48×10^{-7}
$T_R^2\ Q_{gas}$	5.05	0.0340	-2.08×10^{-3}	-1.89×10^{-9}
LOF	2.09	0.1848		

Table S5. ANOVA data of the design concerning the N-CNTs' Fe-content. For each polynomial term the *F* and *p* value is shown as well as the coded [−1/+1] and actual values of the respective coefficients. The used transformation for the fit is given by λ.

Term	*F* value	*p* value	coded	actual
$\lambda = 0.5$				
Model	115.39	<0.0001	1.53	32.43
T_R	809.76	<0.0001	−1.13	-3.89×10^{-2}
Q_I	26.55	<0.0001	0.20	4.62×10^{-1}
Q_{gas}	1.83	0.1905	−0.05	3.37×10^{-3}
$\omega_{pyridine}$	3.44	0.0776	0.07	4.14×10^{-2}
$T_R\ Q_I$	3.60	0.0716	−0.08	-3.18×10^{-4}
$T_R\ \omega_{pyridine}$	24.57	0.001	−0.21	4.62×10^{-5}
$Q_I\ Q_{gas}$	10.02	0:0047	−0.13	-4.83×10^{-4}
T_R^2	31.05	<0.0001	0.36	3.63×10^{-5}
LOF	1.58	0.3213		

S4. Inhomogeneity within samples

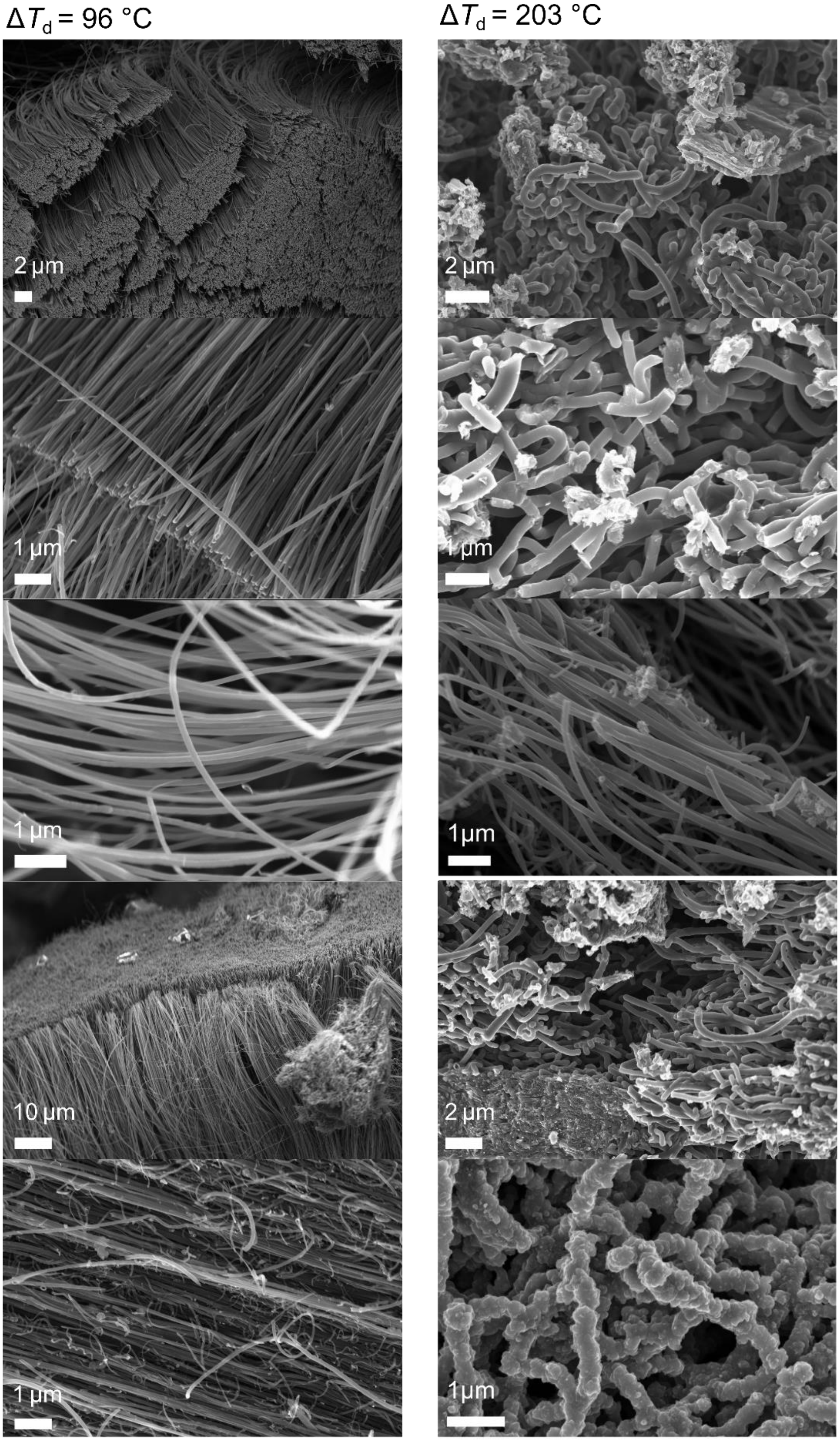

Figure S5. SEM-images illustrating the inhomogeneity within two N-CNT samples with different decomposition interval, ΔT_d. Different positions within the sample can exhibit different morphologies. Sample specifications: $\omega_{pyridine}$ = 22 wt%, Q_I = 10.5 mL h^{-1}, Q_{gas} = 110 mL min^{-1}, T_R = 820 °C (*left*); $\omega_{pyridine}$ = 95 wt%, Q_I = 10.5 mL h^{-1}, Q_{gas} = 330 mL min^{-1}, T_R = 960 °C (*right*).
